# Phytochemical Profiling and Antioxidant Capacity of Traditional Plants, Northern Thailand

**DOI:** 10.3390/plants12233956

**Published:** 2023-11-24

**Authors:** Peerapong Jeeno, Sumed Yadoung, Pichamon Yana, Surat Hongsibsong

**Affiliations:** 1 School of Health Sciences Research, Research Institute for Health Sciences, Chiang Mai University, Chiang Mai 50200, Thailand; peerapong_jeen@cmu.ac.th (P.J.); pichamon_yana@cmu.ac.th (P.Y.); 2 Environment, Occupational Health Sciences and Non-Communicable Disease Center of Excellence, Research Institute for Health Sciences, Chiang Mai University, Chiang Mai 50200, Thailand; sumed_y@cmu.ac.th; 3 Environmental Science Program, Faculty of Science, Chiang Mai University, Chiang Mai 50200, Thailand

**Keywords:** phytochemical, bioactive, traditional plant, northern Thailand, LC-QTOF/MS

## Abstract

Traditional plants have played a significant role in human culture and medicine throughout history. These plants have the capability to synthesize a diverse range of chemical compounds that serve essential biological functions. This study’s objective was to analyze the phytochemical composition of five traditional plants, namely *Emilia sonchifolia*, *Chloranthus erectus*, *Caesalpinia mimosoides*, *Acacia concinna*, and *Tacca chantrieri*, native to northern Thailand, using LC-QTOF/MS analysis and assess their potential bioactivity through various assays including DPPH radical scavenging activity, ABTS radical scavenging activity, ferric ion reducing antioxidant power, total phenolic compounds, and total flavonoid content. The findings revealed the presence of natural bioactive compounds in each plant extract, which exhibited pharmacological activity. Notably, *Caesalpinia mimosoides* displayed the highest antioxidant capacity across all plant extracts (IC_50_ in DPPH with the methanol extract was 0.03 and 898.18 mg AAE/100 g with the ethanol extract), along with elevated levels of total phenolic and flavonoid content, which showed the highest TFC at 46.79 µgRE/g in the methanol extract. In conclusion, traditional plants possess notable biological constituents and antioxidant properties, suggesting their potential for bioactive applications. Based on these findings, these indigenous plants can serve as a valuable resource in traditional medicine, offering the possibility of uncovering new products with similar capabilities and additional therapeutic attributes worthy of future exploration.

## 1. Introduction

Traditional plants have been utilized for therapeutic purposes by numerous cultures throughout history. They are often referred to as medicinal plants or herbs. These plants are frequently abundant in pharmacologically active natural chemicals that are used to treat a variety of medical ailments [1]. Traditional plant uses for medicine are frequently based on regional knowledge and customs that have been passed down from one generation to the next. From ancient times, many traditional plant treatments have been employed, some of which have been scientifically validated and are still used in contemporary medicine [2]. A wide variety of plant species that have historically been used in regional food culture may be found in northern Thailand. Spices and herbs are high in vitamins, nutrients, and dietary fiber, all of which are useful to our health and provide protection. They contain bioactive pharmaceutical compounds that have antioxidant, anticancer, antihypertensive, and antidiabetic properties [3,4,5,6,7].

Phytochemicals are organic molecules that can operate as a plant’s defensive system against various predators [8]. Medicinal flora’s positive and multifunctional pharmacological activities are fundamentally dependent on its phytochemical components [9].

*Emilia sonchifolia*, belonging to the Asteraceae family and commonly known as lilac tassel flower, is a shrub characterized by a softly hairy stem. Typically, it reaches a height of 20–70 cm and possesses a branched taproot. This plant is commonly found as a weed in grassy areas along roadsides and in dense forests [10]. The notable antioxidant activity observed in *Emilia sonchifolia* leaves may be attributed to the presence of phytochemical compounds like flavonoids and tannins. The antioxidant potential of this plant, stemming from its polyphenolic content, aligns with its traditional medicinal use in treating various medical conditions [4].

*Chloranthus erectus* (Buch. -Ham.) Verdcourt, a shrub belonging to the Chloranthaceae family, is found in shaded habitats, close to streams, and on the forest floor. Its distribution spans the tropical and temperate zones of the Eastern Himalayas, Indo-Burma, and the Southeast Asian region [11]. *C. erectus* is a well-known traditional remedy among tribes in India. It is utilized to address specific issues such as localized swelling, joint discomfort, skin inflammation, wound recovery, fever, and bodily pains [12].

*Caesalpinia mimosoides* Lam., a small, spiny, and trailing shrub, is indigenous to the Indian subcontinent and Southeast Asia. It belongs to the Fabaceae family, specifically the Caesalpinioideae subfamily [6]. In Thailand, it is commonly consumed as fresh vegetables and appetizers [13]. The plant has been documented to display antioxidant properties, antimicrobial effects, anti-inflammatory attributes, and anticancer potential [6,13].

*Acacia concinna* DC., known as ‘Som Poy’ in Thai, is also commonly referred to as ‘Som Kon’. This plant is a spiny, climbing shrub typically found in tropical forests. Its leaves are bipinnate and feature clusters of yellow, spherical auxiliary flowers. As the plant’s pods dry, they take on a brown color and develop wrinkles [14]. The plant possesses naturally occurring bioactive metabolites with anticoagulant properties that inhibit blood coagulation as well as antiplatelet properties [15]. In India, Myanmar, and Thailand, the leaves are utilized to treat diabetes and various skin conditions [16].

*Tacca chantrieri* André is among the most commonly found species within the Tacca genus, with its distribution spanning the Thailand-IndoChinese Peninsula and extending to the Malay Peninsula including parts of southern China [17].

Therefore, the aforementioned plants are indigenous and have practical uses in both cooking and medicinal purposes. Furthermore, the plant species *Caesalpinia mimosoides* has good antimicrobial, antioxidant, and wound-healing properties [6]. *Tacca chantrieri* also exhibits analgesic, antipyretic, and anti-inflammatory effects [5], as reported by one study. Consequently, this study aimed to collect essential information on the bioactive compounds that could be highly valuable. To achieve this goal, the comprehensive extraction of plants, namely *Caesalpinia mimosoides*, *Emilia sonchifolia*, *Chloranthus erectus*, *Acacia concinna*, and *Tacca chantrieri,* was carried out to obtain different extracts and then assessed for their polyphenol and flavonoid contents as well as their antioxidant potential. Additionally, the biochemical compounds within the extracts were analyzed using LC-QTOF/MS. This analysis serves as a reference for future investigations into potentially beneficial bioactive compounds.

## 2. Results

### 2.1. Antioxidant Capacity

To assess the antioxidant capacity, we conducted experiments involving the determination of reducing power, DPPH radical scavenging activity, and ABTS radical scavenging activity. Table 1 demonstrates the antioxidant capabilities of the traditional plants. *Emilia sonchifolia* exhibited DPPH scavenging activity with IC_50_ values of 6.98 ± 0.93 mg/mL in the ethanol extract, 2.48 ± 0.74 mg/mL in the methanol extract, and 96.03 ± 24.00 mg/mL in the water extract. Additionally, for ABTS radical scavenging activity, the IC_50_ value was 27.39 mg/mL in the ethanol extract, 32.68 mg/mL in the methanol extract, and 34.54 mg/mL in the water extract. In terms of ferric ion reducing antioxidant power (measured in mg AAE/100 g), the results were 126.69 ± 15.05 in the ethanol extract, 101.66 ± 1.75 in the methanol extract, and 0.17 ± 0.11 in the water extract.

DPPH scavenging activity was demonstrated by *Chloranthus erectus*, with IC_50_ values of 6.21 ± 1.09 mg/mL in the ethanol extract, 4.00 ± 1.78 mg/mL in the methanol extract, and 246.70 ± 71.60 mg/mL in the water extract. Furthermore, the IC_50_ values for ABTS radical scavenging activity in the ethanol extract were 45.18 mg/mL, 53.68 mg/mL in the methanol extract, and 19.88 mg/mL in the water extract. In terms of ferric ion reduction antioxidant strength (measured in mg AAE/100 g), the ethanol extract was 450.09 ± 41.29, the methanol extract was 445.17 ± 33.23, and the water extract was 0.23 ± 0.13.

*Caesalpinia mimosoides* displayed DPPH scavenging activity with IC_50_ values of 0.03 ± 0.03 mg/mL in the methanol extract and 0.03 ± 0.03 mg/mL in the water extract. However, the IC_50_ value could not be determined in the ethanol extract. Furthermore, for ABTS radical scavenging activity, the IC_50_ values were 93.20 mg/mL in the ethanol extract, 97.12 mg/mL in the methanol extract, and 71.21 mg/mL in the water extract. In terms of ferric ion reducing antioxidant power (measured in mg AAE/100 g), the results were 898.18 ± 0.00 in the ethanol extract, 547.10 ± 0.00 in the methanol extract, and 20.24 ± 1.14 in the water extract.

*Acacia concinna* exhibited DPPH scavenging activity with IC_50_ values of 41.32 ± 2.00 mg/mL in the ethanol extract, 25.25 ± 6.56 mg/mL in the methanol extract, and 471.66 ± 4.11 mg/mL in the water extract. Additionally, for ABTS radical scavenging activity, the IC_50_ values were  30.29  mg/mL in the ethanol extract, 78.43 mg/mL in the methanol extract, and 64.26 mg/mL in the water extract. In terms of ferric ion reducing antioxidant power (measured in mg AAE/100 g), the results were 129.59 ± 16.05 in the ethanol extract, 67.86 ± 0.53 in the methanol extract, and 2.07 ± 0.02 in the water extract.

*Tacca chantrieri* demonstrated DPPH scavenging activity with IC_50_ values of 19.74 ± 2.72 mg/mL in the ethanol extract, 12.55 ± 1.46 mg/mL in the methanol extract, and 70.10 ± 62.04 mg/mL in the water extract. Furthermore, for ABTS radical scavenging activity, the IC_50_ values were 45.95 mg/mL in the ethanol extract, 43.22 mg/mL in the methanol extract, and 14.57 mg/mL in the water extract. Regarding ferric ion reducing antioxidant power (measured in mg AAE/100 g), the results were 354.53 ± 5.84 in the ethanol extract, 180.43 ± 11.67 in the methanol extract, and 0.55 ± 0.31 in the water extract.

### 2.2. Total Phenolic Compound and Total Flavonoid Content

**Figure 1:**
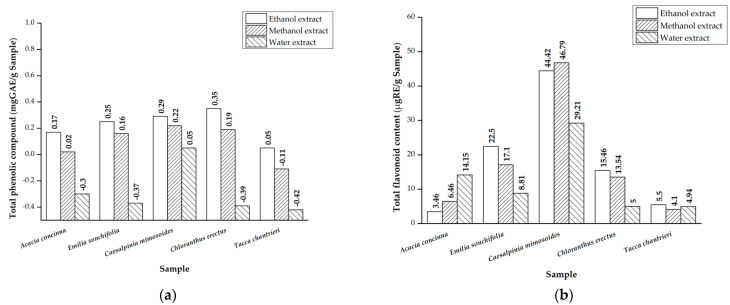
(**a**) Total phenolic compound and (**b**) total flavonoid content.

The content of total phenolic compounds (TPC) and total flavonoid content (TFC) in these traditional plants are presented in Figure 1. The highest TPC values were observed in the ethanol extracts with values of 0.17 mgRE/g in *Acacia concinna*, 0.25 mgRE/g in *Emilia sonchifolia*, 0.29 mgRE/g in *Caesalpinia mimosoides*, 0.35 mgRE/g in *Chloranthus erectus*, and 0.05 mgRE/g in *Tacca chantrieri*. In contrast, the water extracts showed negative results for TPC. The methanol extracts displayed TPC values of 0.02 mgRE/g in *Acacia concinna*, 0.16 mgRE/g in *Emilia sonchifolia*, 0.22 mgRE/g in *Caesalpinia mimosoides*, and 0.19 mgRE/g in *Chloranthus erectus*, but yielded a negative result for *Tacca chantrieri*.

In terms of the total flavonoid content (TFC), *Caesalpinia mimosoides* exhibited the highest TFC in the methanol extract at 46.79 µgRE/g, followed by 44.42 µgRE/g in the ethanol extract and 29.21 µgRE/g in the water extract. *Emilia sonchifolia* showed TFC values of 22.5 µgRE/g in the ethanol extract, 17.142 µgRE/g in the methanol extract, and 8.81 µgRE/g in the water extract. *Chloranthus erectus* had TFC values of 15.46 µgRE/g in the ethanol extract, 13.54 µgRE/g in the methanol extract, and 5.00 µgRE/g in the water extract. *Acacia concinna* displayed the highest TFC value in the water extract at 14.15 µgRE/g, followed by 6.46 µgRE/g in the methanol extract and 3.46 µgRE/g sample in the ethanol extract.

### 2.3. Qualitative Phytochemical Profiling—LC-QTOF/MS

The experimentally measured mass of each ion was matched against the exact mass of the molecular formula and the expected isotope pattern for that formula. These data were used to evaluate the similarity between the ions and the entries in the database through the MassHunter qualitative analysis software’s (Agilent MassHunter Workstation Qualitative Analysis version 10.0, Santa Clara, CA, USA) find-by-formula search of the Agilent MassHunter METLIN Metabolomics Database. Each table presents the top ten compounds from the ethanolic extract of each traditional plant, along with their respective retention times. Table 2 provides the phytochemical profiles of five traditional plants including *Emilia sonchifolia*, *Chloranthus erectus*, *Caesalpinia mimosoides*, *Acacia concinna*, and *Tacca chantrieri*.

## 3. Discussion

### 3.1. Antioxidant Capacity

The results of the total antioxidant capacity, as shown in Table 1, are presented as IC_50_ values for the DPPH radical scavenging activity and ABTS radical scavenging activity using different extraction methods. In the case of *Emilia sonchifolia*, the IC_50_ value for the DPPH scavenging activity was 6.98 ± 0.93 mg/mL in the ethanol extract, whereas a previous study reported an IC_50_ value of 180 µg/mL. The n-hexane extract of *Emilia sonchifolia* showed enhanced free radical scavenging activity in a previous investigation, which could be attributed to the antioxidant activity of the *Emilia sonchifolia* n-hexane extract trapping free radicals by donating hydrogen atoms [18]. Regarding the ferric reducing antioxidant power (FRAP) assay, antioxidants reduce ferric ions (Fe^3+^) to ferrous ions (Fe^2+^). This reduction process is concentration-dependent and results in a color change from yellow to pale green or blue, depending on the concentration of antioxidants in the samples [19]. In the FRAP assay, the ethanol extract exhibited the highest reducing power with a value of 126.69 ± 15.05 mg AAE/100 g sample. Furthermore, for the ABTS radical scavenging activity, the IC_50_ value was 27.39 mg/mL in the ethanol extract. 

In the case of *Chloranthus erectus*, the IC_50_ value for the DPPH scavenging activity was 6.98 ± 0.93 mg/mL in the methanol extract and 4.00 ± 1.78 mg/mL in another study, where the highest antioxidant activity was observed in the methanol extract of both the leaves (88.36 ± 0.24%) and twigs (91.25 ± 0.10%) [20]. Furthermore, the IC_50_ values for ABTS radical scavenging activity in the ethanol extract were 45.18 mg/mL. In terms of ferric ion reducing antioxidant power (measured in mg AAE/100 g ), the ethanol extract exhibited a value of 450.09 mg AAE/100 g.

*Caesalpinia mimosoides* exhibited DPPH scavenging activity with IC_50_ values of 0.03 ± 0.03 mg/mL in the methanol extract and 0.03 ± 0.03 mg/mL in the water extract. However, the IC_50_ value could not be determined in the ethanol extract. In another study, for the ABTS radical scavenging activity, the IC_50_ values were 93.20 mg/mL in the ethanol extract, 97.12 mg/mL in the methanol extract, and 71.21 mg/mL in the water extract. In a previous study, the methanol extract of *Caesalpinia mimosoides* showed antioxidant properties with EC_50_ values of 8.20 ± 0.29 and 5.16 ± 0.98 μg/mL for scavenging free radicals by the DPPH and ABTS assays, respectively [21]; the results suggest that the methanol extract in this study had a high antioxidant property compared to previous study.

For *Acacia concinna*, the IC_50_ value for the DPPH scavenging activity was 25.25 ± 6.56 mg/mL in the methanol extract. Additionally, for the ABTS radical scavenging activity, the IC_50_ value was 30.29 mg/mL in the ethanol extract. In terms of ferric ion reducing antioxidant power (measured in mg AAE/100 g), the highest result was obtained in the ethanol extract with a value of 129.59 ± 16.05. Previous studies have also reported positive antioxidant results for *A. concinna*, with one study indicating a maximum result with distilled water (0.723 ± 0.195 nm) [4] while another study showed significant radical scavenging activity in the DPPH and ABTS assays (IC_50_ of 2.0487 ± 0.1675 mg/mL and 0.5660 ± 0.0033 mg/mL, respectively), suggesting that the aqueous extracts outperformed the ethanolic extract in terms of scavenging activity. As a result, the extracts’ active components may be quite polar or more hydrophilic [22]. A recent study reported a FRAP value of 1016.38 ± 29.29 μmoles TE/100 g sample [23].

For *Tacca chantrieri*, the IC_50_ value for the DPPH scavenging activity was 12.55 ± 1.46 mg/mL in the methanol extract. Furthermore, for ABTS radical scavenging activity, an IC_50_ value of 43.22 mg/mL was also observed in the methanol extract. Regarding the ferric ion reducing antioxidant power (measured in mg AAE/100 g sample), the results were 354.53 ± 5.84 in the ethanol extract. Previous studies have also indicated a high antioxidant activity in *Tacca chantrieri*, particularly in the DPPH radical assay (EC_50_ = 10.24 µg/mL) [24].

### 3.2. Total Phenolic Compound and Total Flavonoid Content

Phenolic compounds encompass a diverse array of molecules including simple flavonoids, phenolic acids, complex flavonoids, and pigmented anthocyanins [25]. These compounds typically consist of one or more aromatic rings with hydroxyl substituents. Common examples of phenolic compounds found in plants include gallic acid, ferulic acid, caffeic acid, coumaric acid, protocatechuic acid, resveratrol, quercetin, and rosmarinic acid [26]. Moreover, numerous phenolic compounds present in traditional plants have been observed to possess antioxidant activity [27].

Regarding the total phenolic compounds of *Emilia sonchifolia*, the ethanol extract had the highest phenolic content (0.25 mgGAE/g), while the water extract showed a negative value (−0.37 mgGAE/g), indicating an absence of phenolic content. Another study reported that the ethyl acetate fraction had the highest phenolic content at 5.804 mg/g [7]. Similarly, the total flavonoid content was the highest in the ethanol extract (22.5 µgRE/g), and another study found the highest total flavonoid content in the ethyl acetate fraction at 10.556 mg/g. However, when compared to the water extract, this study had a TFC value of 8.81 gRE/g, whereas the prior study had no flavonoid content [7].

The total phenolic content (TPC) for the *Chloranthus erectus* samples is presented in Figure 1 and expressed in terms of gallic acid equivalent (mg GAE/g). The highest TPC was recorded for the ethanol extract at 0.35 mg GAE/g sample. In a previous study, the highest TPC was recorded for a methanolic extract of the leaf sample at 9.64 ± 0.15 µg GAE/g dry weight [20]. The total flavonoid content was found to be highest in the ethanol extract (15.46 µgRE/g). In another study, flavonoids were found in the aqueous extract but not in the methanol extract [28]. The amount of the phenolic component in methanolic leaf samples was altered by the solvent polarity due to its polar nature, yielding a larger value [20].

The total phenolic compound (TPC) for the *Caesalpinia mimosoides* samples is presented in Figure 1. The highest TPC was recorded for the ethanol extract at 0.29 mg GAE/g. In a previous study, the highest TPC was recorded for a methanolic extract at 460.25 ± 3.08 mg GAE/g dry weight extract [21]. The total flavonoid content was found to be highest in the methanol extract (46.79 µgRE/g). In another study, flavonoids were found in the methanolic extract at 12.55 ± 0.43 mg GAE/g dry weight extract. The quantities of phenolic compounds and flavonoids differed between investigations, most likely because CM acquired bioactive chemicals from polar rather than nonpolar molecules [21].

Regarding the total phenolic compound content of *Acacia concinna*, the highest TPC was recorded for the ethanol extract at 0.17 mg GAE/g, while the water extract showed a negative value (−0.3 mgGAE/g), indicating an absence of phenolic content. The total flavonoid content was found to be highest in the water extract (14.15 µgRE/g). Another study reported the concentration of phenol (0.2431 ± 0.0002 mg/L) and flavonoid (0.2768 ± 0.0003 mg/L) observed in plant extracts [29].

In terms of the total flavonoid content (TFC), *Tacca chantrieri* exhibited the highest TFC in the ethanol extract at 5.5 µgRE/g. However, for the total phenolic content (TPC), *Tacca chantrieri* showed a result of 0.05 mgGAE/g in the ethanol extract, while the methanol and water extracts displayed negative results. There has been no research on the total phenolic and flavonoid content of *C. erectus* to date. This investigation could be the first step in determining the overall phenolic and flavonoid contents of *C. erectus*.

### 3.3. Qualitative Phytochemical Profiling—LC-QTOF/MS

Humanity has utilized traditional plants for millennia. Many kinds of chemical compounds with significant biological roles can be synthesized from plants. The precise mass of the chemical formula and the anticipated isotope pattern for these formulas were compared to the experimentally determined masses of each ion. Using the Agilent MassHunter METLIN Metabolomics Database’s find-by-formula search, MassHunter qualitative analysis software (Agilent MassHunter Workstation Qualitative Analysis version 10.0, Santa Clara, CA, USA) was utilized to assess the degree of similarity between the ions and the database entries.

The results showed that phytochemical profiling was observed in *Emilia sonchifolia* (L.) DC., as shown in Table 2. All compounds were identified as 2-pyridylacetylglycine, dopexamine, penicillamine cysteine disulfide, dextromoramide, sulfociprofloxacin, 3β,5β-tetrahydronorethindrone glucuronide, pimozide, idebenone metabolite (QS-10), dextroamphetamine, and propranolol. Some compounds are used in drugs such as dopexamine. Dopexamine has been investigated in clinical trials for its diagnostic and treatment potential in conditions like free flap, oral cancer, hypotension, septic shock, and head and neck cancer (Drug Bank). Dopexamine hydrochloride, a synthetic catecholamine similar to dopamine, has been found to reduce afterload, increase renal perfusion, and induce mild cardiac stimulation in both volunteers and heart failure patients [30]. Dextromoramide is an N-acylpyrrolidine compound formed by the formal condensation of pyrrolidine with (3S)-3-methyl-4-(morpholin-4-yl)-2,2-diphenylbutanoic acid. When compared to most other short-acting opioids, dextromoramide has a comparatively low tendency to cause constipation, which is a major issue with opioid analgesics used for cancer pain management. Additionally, tolerance to the analgesic effects develops relatively slowly [31].

The phytochemical profile of *Chloranthus erectus* (Buch. -Ham.) Verdc. is presented in Table 2, which includes the following identified compounds: ondansetron, fluocinolone, 4′-hydroxytamoxifen, talampicillin, levonorgestrel acetate, pimozide, hydroxyprogesterone acetate, bethanechol, idebenone metabolite (QS-10), and dextroamphetamine. Some of these compounds have pharmacological activities such as levonorgestrel acetate, which is a progestin and prodrug of levonorgestrel. Levonorgestrel acetate is also known as 3-ketonorgestimate [32]. Others, like talampicillin, possess antibiotic properties. Talampicillin is a thiazolide carboxylic ester of ampicillin that undergoes hydrolysis in the intestinal mucosa to release free ampicillin [33].

The phytochemical profile of *Caesalpinia mimosoides* Lamk is presented in Table 2 including the following identified compounds: acamprosate, 6-methylthioguanine, L-tyrosine, 3-methoxy-α-methyl-, hydrogen sulfate, penicillamine disulfide, dextromoramide, idebenone metabolite (QS-10), salmeterol, bethanechol, dextroamphetamine, and 3′-HydroxyIndinavir. Some of these compounds have known pharmacological activities such as salmeterol, which is a long-acting β2 adrenergic receptor agonist (LABA) used for managing asthma and symptoms of chronic obstructive pulmonary disease (COPD) [34]. Bethanechol has been reported as potentially effective sialagogues for patients with xerostomia (dry mouth) [35] and acamprosate is approved as an adjunct to psychosocial interventions to support abstinence in alcohol-dependent patients [36].

The phytochemical profile of *Acacia concinna* (Willd.) DC. was observed and is presented in Table 2. This profile includes the identification of various compounds such as senkyunolide M, 1-penten-3-ol, 1-heptadecanol, epanolol, schisanhenol B, tamoxifen N-oxide, misoprostol, pimozide, and stearamide. Epanolol is a novel anti-anginal agent with both beta 1-adrenoceptor antagonist and beta 1-adrenoceptor agonist activity, making it unique. It is primarily metabolized into conjugates before being excreted in urine, and its potential accumulation in elderly patients has been of interest [37]. Misoprostol is a synthetic prostaglandin E1 analog available in oral form, used for preventing and treating gastroduodenal damage induced by nonsteroidal anti-inflammatory drugs (NSAIDs) [38].

The results of phytochemical profiling were observed in *Tacca chantrieri* André, as shown in Table 2. All compounds were identified as 1-heptadecanol, schisanhenol B, tamoxifen N-oxide, dimetacrine, repaglinide, zoapatanol, pimozide, quinestrol, spiroxamine, and stearamide. Dimetacrine is a tricyclic antidepressant (TCA) with imipramine-like effects used in Europe for the treatment of depression [39]. Quinestrol has been used as the estrogen component in menopausal hormone therapy and in combined hormonal birth control. Quinestrol has been used clinically as an oral contraceptive regimen in women [40].

According to the results of this study, the total antioxidant capacity of various plants changes when using different extraction methods. *Emilia sonchifolia* showed the highest DPPH scavenging activity at 6.98 ± 0.93 mg/mL in the ethanol extract, while *Chloranthus erectus* showed the highest antioxidant activity at 126.69 ± 15.05 mg AAE/100 g. *Caesalpinia mimosoides* showed DPPH scavenging activity with IC_50_ values of 0.03 ± 0.03 mg/mL in the methanol extract and 0.03 ± 0.03 mg/mL in the water extract. *Acacia concinna* had the highest DPPH scavenging activity at 25.25 ± 6.56 mg/mL in the methanol extract and the highest ABTS radical scavenging activity at 30.29 mg/mL. *Tacca chantrieri* had the highest DPPH scavenging activity at 12.55 ± 1.46 mg/mL in the methanol extract and the highest ABTS radical scavenging activity at 43.22 mg/mL. The ethanol extract also showed high antioxidant activity, particularly in the DPPH radical assay. Additionally, we analyzed the total phenolic compounds in various plant extracts including *Emilia sonchifolia*, *Chloranthus erectus*, *Caesalpinia mimosoides*, and *Acacia concinna*. The ethanol extract had the highest phenolic content (0.25 mgGAE/g), while the water extract showed a negative value (−0.37 mgGAE/g. The highest total flavonoid content was found in the Methanol extract (46.79 µgRE/g) in *Caesalpinia mimosoides*

Overall, the highest antioxidant activity in this plant extract is due to quillaic acid, a naturally occurring chemical component included in saponins, which is what gives this plant extract its antioxidant effect [41]. This group can be found in *Emilia sonchifolia*, *Chloranthus erectus*, and *Caesalpinia mimosoides*. Their broad variety of biological activities, which include antioxidant capabilities, are well-known. Furthermore, it has been discovered that physiologically active compounds can also have therapeutic effects. For example, bethanechol has been proposed as a potentially helpful sialagogue for patients with xerostomia [35], and this compound can be found in *Chloranthus erectus* and *Caesalpinia mimosoides*. According to these findings, these indigenous plants may prove to be a valuable asset in conventional medicine and may also lead to the development of other products with similar characteristics and other medicinal benefits that merit more research.

## 4. Materials and Methods

### 4.1. Plant Collection

The five plants shown in Table 3 were collected from Ban Hua Thung, Chiang Dao District, Chiang Mai, Thailand on 29–30 December 2022.

### 4.2. Extraction of Plant Sample

Fresh herb leaves were finely chopped and baked at 60 °C for an hour. One gram of the crushed material was extracted with 30 mL of distilled water, 95% ethanol (JT Baker, Phillipsburg, NJ, USA), and 99% methanol (JT Baker, Phillipsburg, NJ, USA). Next, the combinations were kept for three days at room temperature (25 °C). After the soaking period, the samples were agitated at 2500 rpm for 5 min and subsequently filtered using Whatman No. 1 filter paper (Whatman, OH, USA). The filtered samples were concentrated to dryness utilizing a rotary evaporator. The resulting crude extracts were weighed and later reconstituted in dimethyl sulfoxide. Before analysis, the extracted crude material was stored in a refrigerator at 4 °C.

### 4.3. Characterization of Extracted by LC-QTOF-MS

The qualitative dataset of the extracted sample was qualified by using LC-MS in tandem with a quadrupole time-of-flight mass spectrometer (QTOF-MS, Santa Clara, CA, USA), according to the adopted method of [42]. The extracted samples were dissolved in a mixture of 0.01% formic acid and ethanol (1:1, *v*/*v*) at a concentration of 1 mg/mL. Subsequently, a cleanup step was performed using a dispersive solid-phase extraction (SPE) kit designed for fat and pigments (Agilent Technology, Santa Clara, CA, USA). The resulting sample solution was passed through a 0.22 μm filter. The qualitative analysis was carried out by utilizing an Agilent 1290 Infinity II series chromatographic system coupled to a 6546 LC/Q-TOF instrument (Agilent Tech., Santa Clara, CA, USA). This instrument consists of essential components including a degasser, binary pump, column oven, and thermostat-controlled autosampler. The instrument parameters were optimized as follows: for the liquid chromatography (LC) conditions, ultraviolet (UV) detection was set at 330 nm, with a flow rate of 0.2 mL/min and an injection volume of 10 μL. A mobile gradient system was employed, commencing with a composition of 5% acetonitrile (ACN) and 95% water (containing 1% formic acid). The gradient transitioned to 20% ACN over 5 min, followed by further increments to 30% ACN in 5 min, 35% ACN in 5 min, 45% ACN in 5 min, 75% ACN in 5 min, and finally, 95% ACN until the end of the run. Chromatographic separation was achieved using a ZORBAX Eclipse Plus C18 column (2.1 × 150 mm, 1.8 µm). The mass spectrometry (MS) conditions involved electrospray ionization (ESI) in positive mode. The nebulizer operated at 20 psi with a nitrogen (N2) flow rate of 7 L/min. The capillary temperature was maintained at 300 °C, and the flow rate was set at 8 μL/min. The mass-to-charge ratio (*m*/*z*) range was 50–1000, the capillary voltage was set to 4500 V, and the dry heater temperature was maintained at 280 °C. For each ion, the experimentally measured mass was compared to the exact mass of the molecular formula and the expected isotope pattern for that formula. These comparison data were used to assess the similarity between the ions and the entries in the Agilent MassHunter METLIN Metabolomics Database. MassHunter qualitative analysis software (Agilent MassHunter Workstation Qualitative Analysis version 10.0, Santa Clara, CA, USA) was employed to perform a find-by-formula search in the database. The chemical properties and parameters of the compounds were determined using the Medlin Library.

### 4.4. DPPH Radical Scavenging Activity

The free radical scavenging activity of the five plant extracts was determined using [42]. In brief, the sample analysis was conducted using the 1,1-diphenyl-2-picryhydrazyl (DPPH) assay with some minor adjustments. The DPPH stock solution was prepared by dissolving 24 mg of DPPH in 100 mL of methanol. To create a working DPPH solution, 10 mL of the DPPH stock solution was combined with 45 mL of methanol, and the absorbance of this solution was adjusted to 1.1 at 517 nm. Subsequently, 100 µL of the DPPH working solution was mixed with 100 µL of the plant extract solution, which had concentrations ranging from 0.0156 to 1.000 mg/mL, in a 96-well plate. The mixture was then incubated in the dark for 30 min, after which the absorbance was measured at 517 nm using a microtiter plate reader. The DPPH free radical scavenging activity was calculated as a percentage using the following formula: DPPH = [(Acontrol − Asample)/Acontrol] × 100, where Acontrol represents the absorbance reading of the control, and Asample is the absorbance reading of the sample. The IC_50_ value, denoting the sample concentration required to inhibit 50% of DPPH free radicals, was determined graphically by plotting the percentages of DPPH scavenging activity against the sample concentration.

### 4.5. ABTS Radical Scavenging Activity

ABTS was used to determine the antioxidative potential based on the method described by [42]. In brief, the ABTS reagent, employing 2,2-azino-bis (3-ethylbenzothiazoline-6-sulfonic acid) di-ammonium salt as a stable radical in an aqueous solution, was used along with Trolox as a standard. This solution appears green, and its absorbance was measured at a wavelength of 734 nm to determine the relationship between % inhibition ABTS and sample concentration. To generate ABTS radicals, a reaction was initiated between 7 mM ABTS in water and 2.45 mM potassium persulfate. This mixture was stored in the dark at room temperature for 12–16 h before usage. Subsequently, the ABTS solution was diluted with 80% ethanol to achieve an absorbance value of 0.7 ± 0.02 at 734 nm. For the assay, 190 µL of the ABTS working solution was added to a 96-well plate, followed by the addition of 10 µL of the plant extract, which had concentrations ranging from 0.0156 to 1.000 mg/mL. After an incubation period of 10 min in the dark, the absorbance was measured at 734 nm. Each assay included an appropriate solvent blank. These measurements were repeated at least three times. The percent inhibition of absorbance at 734 nm was calculated using the formula: ABTS radical scavenging effect (%) = [(Aab − Aa)/Aab] × 100, where Aab represents the absorbance of ABTS radical + methanol, and Aa is the absorbance of ABTS radical + sample extract/standard. Trolox served as the standard substance.

### 4.6. Ferric Ion Reducing Antioxidant Power

FRAP was determined using [42]. In brief, to prepare the FRAP (ferric reducing antioxidant power) reagent, a mixture of 2.5 mL of 10 mM TPTZ (2,4,6-tripyridyl-s-triazine) solution in 40 mM HCl, 2.5 mL of 20 mM ferric chloride (FeCl_3_), and 25 mL of 300 mM acetate buffer at pH 3.6 was prepared. For the assay, each gram of the plant extract solution was diluted by a factor of two with distilled water. Subsequently, 10 µL aliquots of the plant extract solution were combined with 190 µL of the FRAP reagent in a 96-well microtiter plate. The mixture was left to incubate in the dark for 30 min, after which the absorbance of the sample was measured at 593 nm using a microtiter plate reader. Ascorbic acid was used to create the standard curve, covering a concentration range of 3.125–200 µg/mL. The FRAP value for each sample was determined by comparing it to the standard curve. The results were expressed as milligrams of ascorbic acid equivalent per 100 g of plant extract (mg AAE/100 g).

### 4.7. Total Phenolic Compound

The total phenolic compound was determined using the Folin–Ciocalteu method, which was modified by [43]. In brief, in this method, gallic acid (Fluka) is employed as the standard substance. Consequently, the total phenolic content is reported in milligrams of gallic acid per gram of sample weight. To establish the standard curve, different concentrations of gallic acid (0.02, 0.04, 0.08, 0.16, 0.32, and 0.64 mg/mL) were prepared in 80% methanol by mixing 12.5 µL of the sample with 12.5 µL of the Folin–Ciocalteu solution (MERCK, Darmstadt, Germany), which had been diluted tenfold with distilled water. After 6 min, 125 µL of 7% sodium carbonate (Na_2_CO_3_) and 100 µL of distilled water were added to the mixture, and the reaction was allowed to proceed at room temperature for 90 min. Subsequently, the absorbance was measured at 760 nm using a microplate reader (SPECTROstar Nano, Ortenberg, Germany). The phenolic concentration was quantified using calibration curves, and the results were expressed in milligrams of gallic acid equivalents per gram of sample (mg GAE/g sample).

### 4.8. Total Flavonoid Content

The total flavonoid content was measured by a colorimetric assay described previously by [44] with slight modifications. A total of 25 µL of the sample solution was put into a 96-well plate, and 7.5 µL 7% NaNO_2_ solution (LOBA CHEMIE PVT. LTD, Mumbai, India) and 12.5 µL distilled water were added and mixed thoroughly. The solution was allowed to stand at room temperature for 5 min. Next, 15 µL 10% AlCl_3_ solution (QRëC) was added to the flask, mixed well, and kept at room temperature for 5 min. Finally, 50 µL of 1 M NaOH solution (ACI Labscan, Bangkok, Thailand) and 27.5 µL distilled water were added, mixed well, and kept at room temperature for 5 min. Absorbance at 510 nm was measured against the water blank using a microplate reader (SPECTROstarNano), and the concentration of flavonoids was estimated using calibration curves. Results were expressed as “ug of quercetin equivalents per g of sample” (ug QE/g sample).

## 5. Conclusions

In this study, different extracts (ethanol, methanol, and water) of traditional plants, namely *Emilia sonchifolia*, *Chloranthus erectus*, *Caesalpinia mimosoides*, *Acacia concinna*, and *Tacca chantrieri,* were examined for their potential activity and their phenolic and flavonoid compounds. Among the methanol extracts, *Caesalpinia mimosoides* displayed the highest antioxidant capacity, accompanied by elevated levels of total phenolic and total flavonoids. Notably, the methanol extract exhibited the highest total flavonoid content. These findings suggest that these native plants hold promise as valuable resources in the field of medicine. For instance, the naturally occurring chemical component quillaic acid, which provides this plant extract with its antioxidant activity. *Caesalpinia mimosoides* contains acamprosate, which could be utilized in combination with psychosocial interventions to support abstinence in alcohol-dependent patients. Additionally, compounds like bethanechol, found in both *Caesalpinia mimosoides* and *Chloranthus erectus*, have the potential to serve as effective sialagogues for individuals with xerostomia. This research lays the foundation for further exploration of the biological value of these plants in future studies.

## Figures and Tables

**Table 1:** DPPH scavenging activity, ABTS radical scavenging activity, and ferric ion reducing antioxidant power of the extract of traditional plants.

Sample	Extraction	Method		
		DPPH-Scavenging Activity	ABTS Radical Scavenging Activity	Ferric Ion Reducing Antioxidant Power
		IC_50_	IC_50_	mg AAE/100 g
*Emilia sonchifolia*	Ethanol	6.98 ± 0.93	27.39	126.69 ± 15.05
	Methanol	2.48 ± 0.74	32.68	101.66 ± 1.75
	Water	96.03 ± 24.00	34.54	0.17 ± 0.11
*Chloranthus erectus*	Ethanol	6.21 ± 1.09	45.18	450.09 ± 41.29
	Methanol	4.00 ± 1.78	53.68	445.17 ± 33.23
	Water	246.70 ± 71.60	19.88	0.23 ± 0.13
*Caesalpinia mimosoides*	Ethanol	ND	93.20	898.18 ± 0.00
	Methanol	0.03 ± 0.03	97.12	547.10 ± 0.00
	Water	0.42 ± 0.15	71.21	20.24 ± 1.14
*Acacia concinna*	Ethanol	41.32 ± 2.00	30.29	129.59 ± 16.05
	Methanol	25.25 ± 6.56	78.43	67.86 ± 0.53
	Water	471.66 ± 4.11	64.26	2.07 ± 0.02
*Tacca chantrieri*	Ethanol	19.74 ± 2.72	45.95	354.53 ± 5.84
	Methanol	12.55 ± 1.46	43.22	180.43 ± 11.67
	Water	70.10 ± 62.04	14.57	0.55 ± 0.31

**Table 2:** Phytochemical profiling of five traditional plants.

No.	Compounds Name	Molecular Formulas	RT	Matching Score (%)	*m*/*z*	Mass	Plants
1	2-Pyridylacetylglycine	C_9_H_10_N_2_O_3_	0.903	95.92	212.1033	194.0694	*Emilia sonchifolia*
2	Dopexamine	C_22_H_32_N_2_O_2_	1.171	96.6	357.2538	356.2461	*Emilia sonchifolia*
3	Penicillamine cysteine disulfide	C_8_H_16_N_2_O_4_S_2_	1.255	93.74	244.1701	243.1630	*Emilia sonchifolia*
4	Dextromoramide	C_25_H_32_N_2_O_2_	1.672	95.07	415.2346	392.2453	*Emilia sonchifolia*, *Caesalpinia mimosoides*
5	Sulfociprofloxacin	C_17_H_18_FN_3_O_6_S	2.040	94.92	429.1240	411.0901	*Emilia sonchifolia*
6	3β,5β-Tetrahydronorethindrone glucuronide	C_26_H_38_O_8_	4.13	93.87	479.2624	478.2553	*Emilia sonchifolia*
7	Pimozide	C_28_H_29_F_2_N_3_O	4.13	97.97	479.2624	461.2287	*Emilia sonchifolia,* *Chloranthus erectus,* *Acacia concinna,* *Tacca chantrieri*
8	Idebenone metabolite (QS-10)	C_19_H_28_O_6_	7.992	95.77	370.2226	352.1887	*Emilia sonchifolia,* *Chloranthus erectus,* *Caesalpinia mimosoides*
9	Dextroamphetamine	C_9_H_13_N	14.229	99.35	136.1122	135.1049	*Emilia sonchifolia*, *Chloranthus erectus,* *Caesalpinia mimosoides*
10	Propranolol	C_16_H_21_NO_2_	14.814	96.63	260.1651	259.1578	*Emilia sonchifolia*
11	Quillaic acid	C_30_H_46_O_5_	6.48	71.9	509.3257	486.3354	*Emilia sonchifolia,* *Chloranthus erectus,* *Caesalpinia mimosoides*
12	Ondansetron	C_18_H_19_N_3_O	0.273	96.16	311.1868	293.1528	*Chloranthus erectus*
13	Fluocinolone	C_21_H_26_F_2_O_6_	1.477	98.32	412.1696	461.2287	*Chloranthus erectus* *Chloranthus erectus*
14	4′-Hydroxytamoxifen	C_26_H_29_NO_2_	1.477	93.78	388.2278	387.2211	*Chloranthus erectus*
15	Talampicillin	C_24_H_23_N_3_O_6_S	1.678	96.79	499.1647	481.1310	*Chloranthus erectus*
16	Levonorgestrel acetate	C_23_H_30_O_3_	3.50	98.04	355.2271	354.2195	*Chloranthus erectus*
17	Hydroxyprogesterone acetate	C_23_H_32_O_4_	4.637	96.31	395.2197	372.2302	*Chloranthus erectus*
18	Bethanechol	C_7_H_17_N_2_O_2_	9.503	95.77	179.1625	161.1293	*Chloranthus erectus,* *Caesalpinia mimosoides*
19	Acamprosate	C_5_H_11_NO_4_S	1.08	97.78	204.0303	181.0410	*Caesalpinia mimosoides*
20	6-Methylthioguanine	C_6_H_7_N_5_S	1.097	93.33	204.0393	352.1888	*Caesalpinia mimosoides*
21	L-Tyrosine, 3-methoxy-a-methyl-, hydrogen sulfate	C_11_H_15_NO_7_S	1.113	95.46	323.0896	305.0558	*Caesalpinia mimosoides*
22	Penicillamine disulfide	C_10_H_20_N_2_O_4_S_2_	1.464	92.49	319.0748	296.0857	*Caesalpinia mimosoides*
23	Salmeterol	C_25_H_37_ NO_4_	8.888	95.51	438.2620	415.2727	*Caesalpinia mimosoides*
24	3′-HydroxyIndinavir	C_36_H_47_N_5_O_5_	14.84	97.33	647.3916	629.3573	*Caesalpinia mimosoides*
25	Senkyunolide M	C_16_H_22_O_4_	0.794	70.52	301.1417	278.1523	*Acacia concinna*
26	1-Penten-3-ol	C_5_H_10_O	0.899	86.65	104.1065	86.0727	*Acacia concinna*
27	1-Heptadecanol	C_17_H_36_O	0.939	82.27	274.311	256.2776	*Acacia concinna*, *Tacca chantrieri*
28	Epanolol	C_20_H_23_N_3_O_4_	1.480	78.63	387.2027	369.1691	*Acacia concinna*
29	Schisanhenol B	C_22_H_26_O_6_	1.482	97.63	409.1629	135.1050	*Acacia concinna*, *Tacca chantrieri*
30	Tamoxifen N-oxide	C_26_H_29_NO_2_	1.483	64.06	410.22107	387.2214	*Acacia concinna*, *Tacca chantrieri*
31	Misoprostol	C_22_H_38_O_5_	2.984	70.91	405.2618	382.2726	*Acacia concinna*
32	Stearamide	C_18_H_37_NO	13.269	85.02	284.2954	283.2881	*Acacia concinna*, *Tacca chantrieri*
33	Dimetacrine	C_20_H_26_N_2_	1.547	95.21	295.2171	294.2101	*Tacca chantrieri*
34	Repaglinide	C_27_H_36_N_2_O_4_	1.678	77.29	453.2759	452.2687	*Tacca chantrieri*
35	Zoapatanol	C_20_H_34_O_4_	2.994	76.26	405.2618	338.2458	*Tacca chantrieri*
36	Quinestrol	C_25_H_32_O_2_	4.285	70.90	382.2756	364.2417	*Tacca chantrieri*
37	Spiroxamine	C_18_H_35_NO	13.145	84.43	320.2565	297.2673	*Tacca chantrieri*

**Table 3:** The traditional plant species analyzed in this study.

Local Name	Scientific Names (Family Names)	Medicinal Properties	
Pahang	*Emilia sonchifolia* (L.) DC.	Anti-inflammatory and analgesic potential, antioxidant [7]	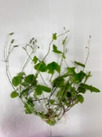
Praya sam sib song mia	*Chloranthus erectus* (Buch. -Ham.) Verdc.	Anti-inflammatory [11], antipyretic, and antibacterial [3]	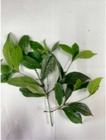
Phak puya, Nam puya	*Caesalpinia mimosoides* Lamk	Antimicrobial, antioxidant, wound healing [6]	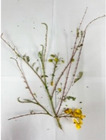
Som Poy	*Acacia concinna* (Willd.) DC.	Antioxidant [4]	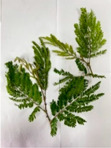
Wan Kang Khaw Dum	*Tacca chantrieri* André	Analgesic, antipyretic, and anti-inflammatory [5]	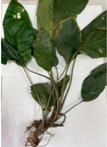

## Data Availability

Data are contained within the article.
